# *De novo* Semi-alignment of 16S rRNA Gene Sequences for Deep Phylogenetic Characterization of Next Generation Sequencing Data

**DOI:** 10.1264/jsme2.ME12157

**Published:** 2013-04-20

**Authors:** Ekaterina Avershina, Trine Frisli, Knut Rudi

**Affiliations:** 1 Norwegian University of Life Sciences, Department of Chemistry, Biotechnology and Food Science, P.O. Box 5003, 1432 Ås, Norway; 2 Hedmark University College, Faculty of Education and Natural Sciences, P.O. Box 4010, 2306 Hamar, Norway

**Keywords:** 16S rRNA gene, multimers, semi-alignment, pyrosequencing

## Abstract

We addressed the challenges of analyzing next-generation 16S rRNA gene deep sequencing data from the uncharacterized microbial majority. This was performed using a novel *de novo* semi-alignment approach. The semi-alignments were based on Orthologous Tri-Nucleotides (OTNs), which are identical trinucleotides located in the same sequence region. OTNs in high error homopolymeric tracts were excluded to avoid overestimation of genetic distances. Phylogenetic information was derived assuming an exponential decay in shared OTNs between pairs of bacteria. OTN relatedness was also explored through principal component analysis (PCA). In evaluating the OTN approach we reanalyzed a dataset consisting of triplicate GS FLX titanium pyrosequencing runs for each of two experimental soil samples, in addition to analyses of the Greengenes core dataset. The conclusion from these comparisons was that the OTN approach was superior to traditional alignments both with respect to speed and accuracy. We therefore believe that our OTN-based semi-alignment approach will be a valuable contribution to future exploration of deep sequencing data.

New in-depth DNA sequencing tools (8) open the possibility for characterizing the hidden microbial biosphere (16). There are, however, several hurdles that have to be overcome. The main challenges are that most of the current analytical approaches are based on some type of predefined alignment or classification model. Microorganisms not covered by the models would therefore be poorly characterized, potentially leading to both type I and II errors. Habitats such as soil, in which most of the microorganisms are not yet characterized, could therefore lead to erroneous interpretations. The main reason for using model-based approaches is that these are generally less computational demanding than the *de novo* methods. Therefore, more computationally efficient *de novo* analyses could potentially increase our understanding of as yet uncharacterized micro-organisms since no or low *a priori* information is needed.

We have previously developed a *de novo* alignment-independent approach denoted Alignment-Independent Bilinear Multivariate Modeling (AIBIMM), addressing the computational complexity of large datasets (11, 12, 15). The principle of AIBIMM is to transform sequences into word frequencies, with subsequent use of word frequencies in ecological and evolutionary comparisons (11, 12). Due to the relatively long match regions required (pentamers), there is a loss of phylogenetic information with this method. The aim of the work presented here was to develop the word-frequency concept further in order to address challenges with sequencing errors, and the retrieval of phylogenetic information. This was performed using a novel high-throughput semi-alignment approach. Semi-alignments were obtained using the concept of Orthologous Tri-Nucleotides (OTNs). OTNs are tri-nucleotides with the same evolutionary origin in different species. Our approach was to use sequence positional information to identify OTNs, where homologous tri-nucleotides located in the same sequence regions were assumed to be OTNs. The OTN identification principle is schematically shown in Fig. 1. Furthermore, we propose a new alignment-independent approach for distance calculations based on OTN’s. Most genes—including those encoding 16S rRNA—are neutrally evolving (7). The divergence rate between the different lineages may, however, vary due to replication and/or mutation rates (10). Independent of the source of divergence, the pair-wise sharing of OTNs between bacteria should approximately follow an exponential decay, as a combined effect of the number of generations separating the bacteria and the mutation rate. The OTN distances calculated by us are based on the exponential decay assumption.

In demonstrating the application of the OTN approach, we chose a recent dataset from an experimental soil microecosystem (6). Soil represents one of the most complex microbial consortia on earth (19); therefore, we believe that soil is a good model for evaluating new deep sequencing *de novo* analytical approaches. In addition, we investigated the well-defined Greengenes core dataset, generated from high-quality Sanger sequences. Our analyses confirmed the better speed and higher resolution of the OTN approach compared to conventional alignments.

## Materials and Methods

### Soil 16S rRNA gene pyrosequencing dataset

We reanalyzed an experimental soil dataset for which the metagenome composition, functionality and size have previously been characterized (6). The dataset represents a simple model system with two experimental soil samples with the same origin, where one sample was exposed to earthworms, while the other was not. Three independent DNA isolations, 16S rRNA gene PCR amplification (V3 and V4 with pyrosequencing tagged forward 5′-TCCTACGGGAGGCAGCAGT-3′ and reverse 5′-GGACTACC AGGGTA-TCTAATCCTGTT-3′ primer with cycling conditions of 95°C for 30s and 60°C for 1 min, using 30 cycles). Pyrosequencing was performed using a 454 GS FLX Titanium instrument at the Norwegian High-Throughput Sequencing Centre (http://www.sequencing.uio.no). Detailed protocols for the analyses are provided in Frisli *et al.* (6). The data have been deposited at www.umb.no/statisk/midivlab/Soilmetagenome.zip.

### OTN semialignment

The sequences were first trimmed and quality checked using the CLC Genomic Workbench software version 3.7.1 (CLC Bio, Århus, Denmark) using an ambiguous nucleotide threshold of 2, and quality threshold of 0.05. In addition, all sequence reads below 300 nucleotides were discharged. The next step was primer identification and removal. This was performed in the Matlab programming environment (Matlab version 7.10; The MathWorks, Natick, MA, USA). Only sequences with 100% match to the forward and reverse primers were included. To simplify further analyses, the number of reads for each of the samples analyzed was reduced to the sample with the lowest number, which was 8,000 reads.

Histogram plots were generated for the complete dataset where the frequencies of all 64 possible trinucleotides were determined relative to the respective sequence positions. The histogram peaks were identified using the Matlab function Peakdet (billauer.co.il/peakdet.html). OTNs were subsequently identified based on binning with respect to the identified peaks in the histogram plots. If at least one sequence contained more than one occurrence of a trinucleotide within a peak, then that trinucleotide position was discharged for further analysis. Finally, the OTN data were tabulated as 1 if present and as 0 if absent for each taxon.

Unique OTN patterns (100% identity) were used as operational taxonomic units (OTUs) in the ecological analyses. Only OTUs observed more than once were considered in the ecological analyses to avoid overestimation of species richness by sequencing errors or chimerical sequences. Species Diversity & Richness software (Pisces Conservation, Hampshire, UK) was used to determine α-diversity measures, while EcoStat software (Trinitysoftware) was used to determine β-diversity.

### Conventional alignments

To compare a novel OTN approach to conventional OTU-based analysis, data were analyzed using the QIIME pipeline (2). Sequences were filtered and chimeras were removed using OTUPIPE, which incorporates both the UCHIME algorithm (4) and ChimeraSlayer reference-based method. After quality filtering, chimera removal and normalization, 17,198 sequences were used for subsequent analysis with a mean of 2,866 sequences per sample (ranging from 2,240 to 3,334 sequences per sample). Sequences were clustered at the 100% similarity level, and the RDP classifier (20) was used to assign taxonomic identity to the resulting OTUs.

Alpha-diversity was assessed using Simpson’s and Shannon diversity indices, whereas beta-diversity was evaluated using unweighted UniFrac (9) distance measurement based on 10 rarefactions with 2,100 randomly selected sequences.

### Statistical analyses

Standard statistical analyses were performed using TIBCO S+ 8.1 software (Tibco Software, Palo Alto, CA, USA), SYSTAT 13 (Systat Software, Chicago, IL, USA) and Microsoft Office Excel 2007 (Microsoft Corporation, Redmond, WA, USA). Multivariate statistical analyses were performed using the PLS Toolbox (Eigenvector Research, Wenatchee, WA, USA). We used the PCA module with data centering prior to analysis. The number of components in the PCA model was chosen to cover 50% of the variance in the data.

### Phylogenetic reconstruction

Phylogenetic analyses based on the trinucleotide data were performed using an exponential decay function for the number of shared trinucleotides as a function of the number of generations. Our model assumes an average mutation rate of 0.5×10^−9^ per position per generation for the 16S rRNA gene. This estimate was based on neutral mutation rates and negative selection for the 16S rRNA gene (3, 18). Based on this estimate we calculated the number of generations between each pair of sequences using the following formula:

(Eq. 1) $$gen=\frac{\text{log}(shared)-\text{log}(total)}{\text{log}(1-rate)}$$

*gen* is the number of generations separating a pair of sequences, *shared* is the number of shared trinucleotides, *total* is the sum of trinucleotides for both sequences, and *rate* is the trinucleotide mutation rate per generation. The support for each branch was tested by bootstrapping with 100 replicates using a MATLAB script.

Alignment-based hierarchical clustering trees were constructed using the neighbor-joining algorithm (13) with CLUSTAL W-generated alignments (17). The analyses were performed by the bioinformatics module in MATLAB.

### Comparison of OTN and conventional alignment-based analyses to high-quality Sanger sequencing data

For evaluations on well-defined high quality Sanger sequences we used the Greengenes core dataset. This dataset was downloaded from greengenes.lbl.gov. One thousand sequences from the dataset were chosen for analysis to avoid extensive computer usage.

In order to evaluate the effectiveness in resolving deep branches, we compared all possible pairwise comparisons for our OTN approach with that obtained for Smith Waterman local alignments. The results’ evaluations were based on histogram plots. The analyses were performed using the bioinformatics module in MATLAB.

We also compared the concordance with taxonomic assignments at the phylum level for OTN and conventional alignments since deep phylogenetic lineages are difficult to resolve. Phylum level taxonomic assignments were determined using the naïve Bayesian classifier in the Ribosomal Database Hierarchical Classifier (20). The traditional alignments were obtained using the alignment module in CLC Genomic Workbench (CLC Bio, Århus, Denmark). The aligned data were subsequently transformed to binary data where the presence and absence of the four nucleotides, in addition to gaps, were coded as 1 and 0. Both the OTN and the binarized conventional alignment data were analyzed by PCA. Finally, the concordances between taxonomic assignments and placements in the PCA plots were determined by ANOVA using SYSTAT 13 software.

## Results

### Identification of OTNs from the soil pyrosequencing dataset

**Fig. 2:**
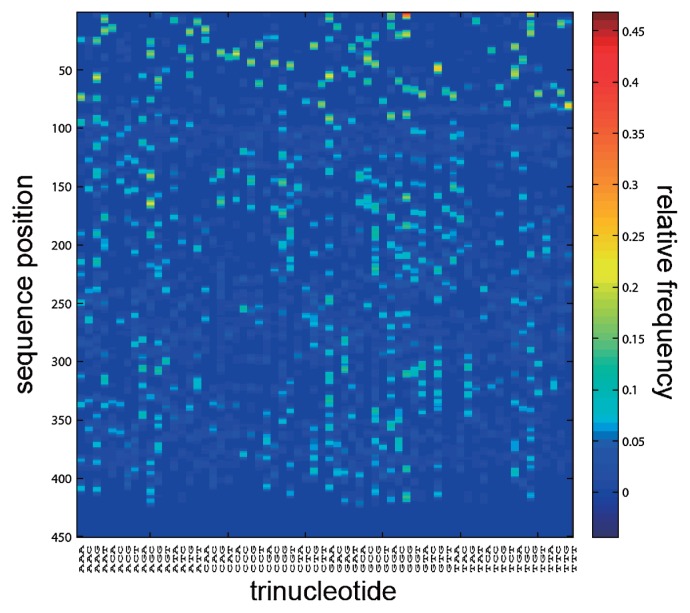
Trinucleotide positional dependence. The relative frequency of each of the 64 possible trinucleotides at each sequence position is shown. The frequencies represent a summary of the complete dataset analyzed here.

**Fig. 3:**
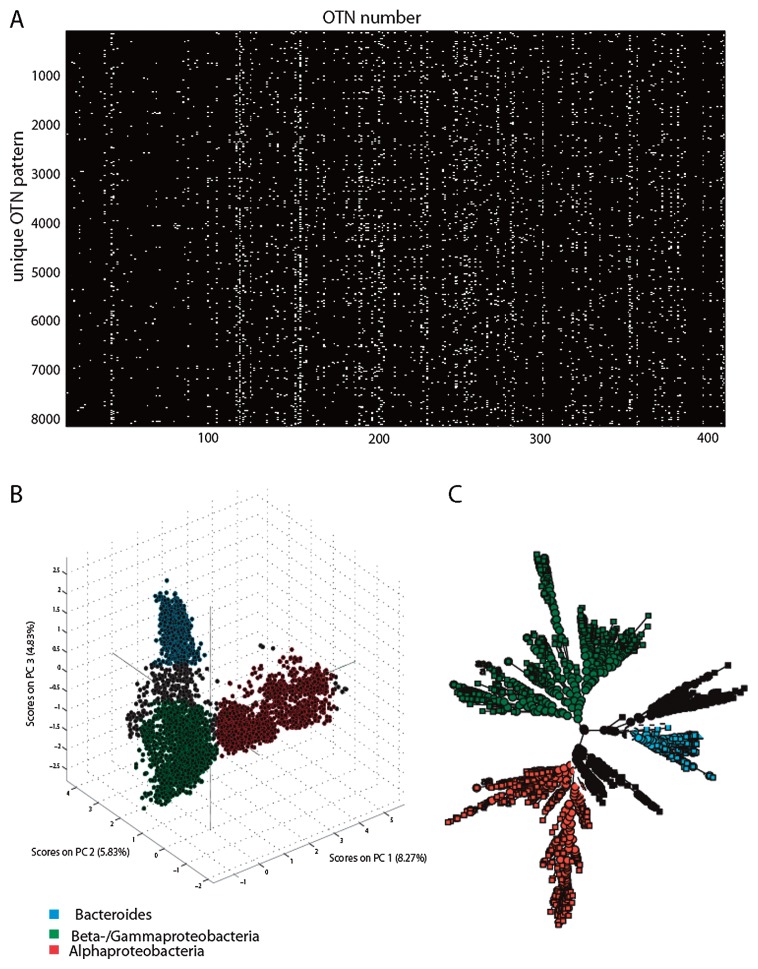
OTN patterns identified. (A) All the OTN patterns identified in the current work are shown with the color-coding of black absence and white presence. Only OTNs with prevalence >10 are shown. (B) Coordinate clustering, and (C) hierarchical clustering of PCA compressed data. The hierarchical clustering is based on a random selection of 1,000 OTN patterns due to computational complexity.

Our first analysis was to identify the overall position dependence of the trinucleotides. This was performed by determining the average frequency of all 64 trinucleotides at all sequence positions for all the sequences combined. These analyses showed clearly distinct non-random distribution patterns (Fig. 2). Based on the histogram patterns, 740 unique peaks were identified. The trinucleotides in different species were considered as OTNs. We identified 8072 OTN patterns (Fig. 3A), of which 7042 were represented by only a single sequence. OTN patterns showed three main clusters (Fig. 3B and C). RDP hierarchical classifier assignments of these clusters to conventional taxonomy (based on selected sequences due to computational complexity) suggested that the main clusters represent the phylum *Bacteroides*, and the classes *Betaproteobacteria*, *Gammaproteobacteria*, and *Alphaproteobacteria*.

### OTN-based phylogenetic reconstruction and validation

**Fig. 4:**
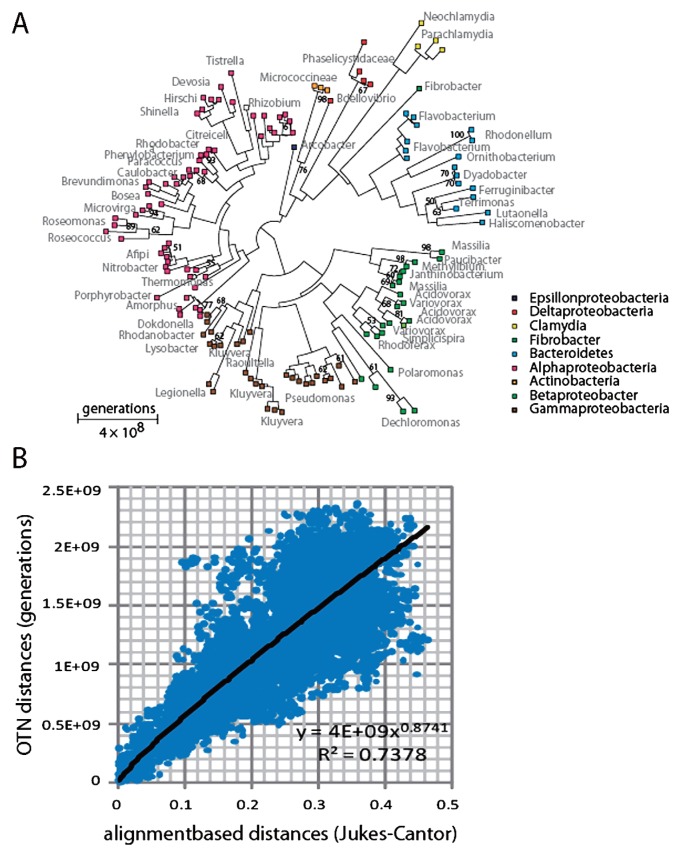
OTN-based phylogenetic reconstruction. (A) The 143 most abundant sequence-types were selected for OTN distance-based phylogenetic reconstruction. The numbers at the nodes indicate the percent bootstrap support (>50%). (B) Regression between OTN-and Jukes-Cantor alignment-based distances. The alignments were generated by Clustal W.

The most dominant OTN patterns were selected for validation of the OTN-based phylogenetic reconstruction approach. These analyses confirmed the dominance of *Bacteroides*, *Betaproteobacteria*, *Gammaproteobacteria* and *Alphaproteobacteria*, but revealed additional phyla such as *Actinobacteria* and *Chlamydia* (Fig. 4A). The generation calculations suggest that the maximum distance is about 5×10^9^ generations among the most distantly related phyla in our dataset.

In order to validate the phylogenetic information, the OTN distances were compared to that of distances derived from traditional DNA sequence alignments. These analyses showed good correspondence between the two distance calculations (Fig. 4B). The relation, however, was not completely linear. The alignment-based distances were slightly larger for long distances.

### Diversity measures

Due to the potential of either representing sequencing errors or chimerical sequences, OTN patterns that were only encountered once were not included in the diversity measurement analyses. Assuming that the singletons represent sequencing errors, then the per-position accuracy rate was 99.8% for our dataset.

The OTN α-diversity measures indicated very high diversity (Table 1), with higher α-diversity in the non-earthworm sample, as compared to the samples containing earthworms. This was determined by the Mann-Whitney U test for both Shannon-Wiener H and Simpson’s D indexes (*p*=0.05 for both). For the conventional OTU-based analyses there were also significant differences between the earthworm and non-earthworm samples in both the Simpson’s D (*p*=0.05), and the Shannon-Wiener H (*p*=0.05). The overall higher diversity in OTN, as compared to conventional OTU analyses, can probably be explained by error filtering criteria.

**Fig. 5:**
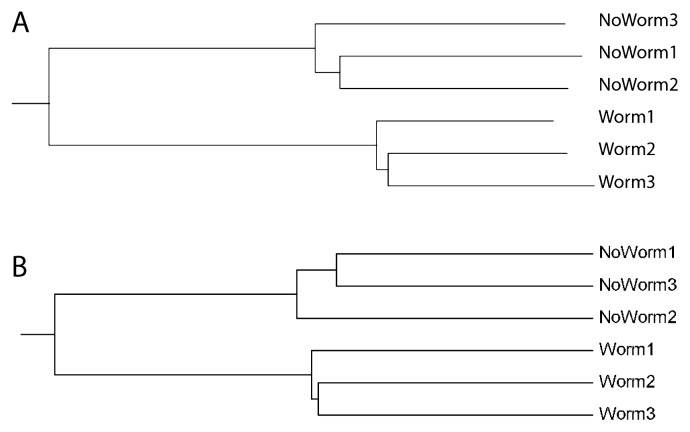
Comparison of OTN semi-alignment (A) and conventional alignment-based (B) beta diversity measures for the six libraries analyzed. (A) Chord distances between the six libraries included based on the semi-alignment approach. (B). Alignment-based unweighted Unifrac diversity measure based on 10 rarefactions with 2,100 sequences used for each rarefaction.

The β-diversity measures showed a clear separation of the two samples analyzed, while the intra-sample diversity was quite low. Similar patterns were revealed by both OTN- and OTU-based analyses (Fig. 5).

### High-quality reference dataset comparisons of OTN and conventional alignments

**Fig. 6:**
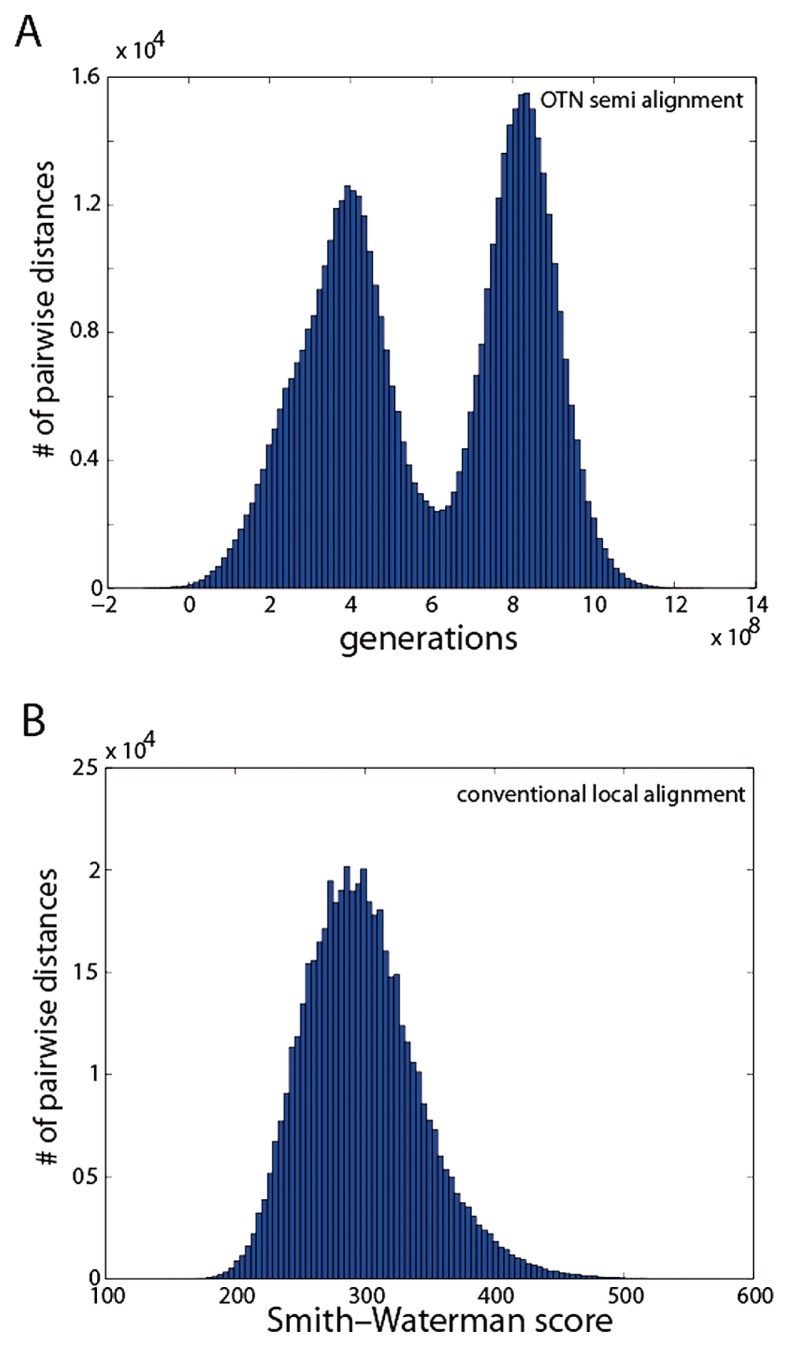
Pairwise semi-alignment (A) and conventional alignment-based (B) distances for the Greengenes reference dataset. The numbers of pairwise distances within the intervals indicated on the abscissa are plotted on the ordinate as histograms with 100 bins.

Finally, we compared the performance of the OTN and conventional alignment approach using the Greengenes reference dataset. Comparisons of pairwise distances suggest that the resolution of the OTN distances is better than for conventional alignment-based distances. The OTN approach gave two clear peaks with respect to pairwise distances, while the alignment-based approach gave only one (Fig. 6). This suggests that there are underlying distance structures not captured by the alignment approach. Furthermore, the speed of the pairwise OTN comparisons was about tenfold that of the conventional alignments.

**Fig. 7:**
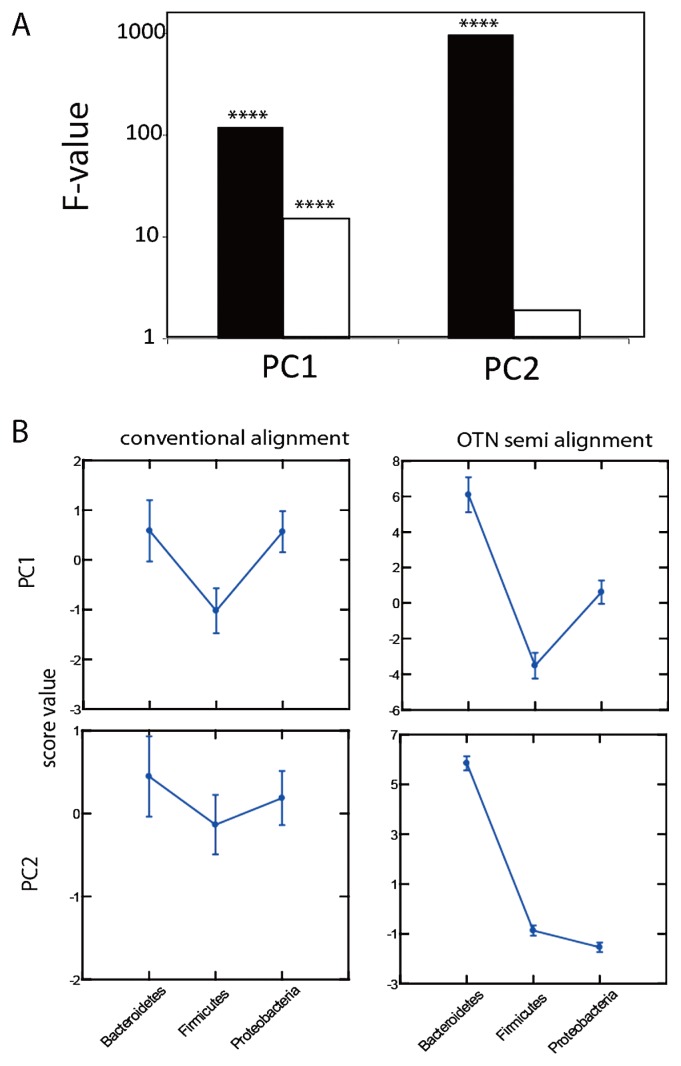
PCA-based separation of the Greengenes reference dataset at the phylum level. (A) Comparison of the concordance between the phylum level classification and PCA coordinate 1 and 2 for OTN semi-alignment (black bars) and conventional multiple sequence alignment (white bars). Concordance is represented with the ANOVA F-value. Significance levels are denoted by *=0.05, **=0.01, ***=0.001 and ****<0.001. (B) Least square means with standard error bars for the three most dominant phyla for conventional alignment and OTN semi-alignment.

The increased resolution for deep phylogenetic branches was also confirmed by PCA analyses. Here we investigated the concordance between phylum level classification of the reference sequences and placement of the PCA coordinates. Clearly, the OTN approach gave much better concordance than the conventional alignments (Fig. 7).

## Discussion

Although the alignment models perform well on known bacterial phylotypes (14), there are inherent risks that uncharacterized phylotypes may align wrongly without being recognized. Using the RDP hierarchical classifier on the dominant phylotypes in our dataset only about eighty-five of the phylotypes were classified to the genus level. Even more alarming, however, was the fact that completely artificial sequences were classified with one hundred percent confidence at the genus level. Since our biological understanding of environmental microorganisms is so low, such errors may go unrecognized as we do not know what to expect. Furthermore, the extensive error and chimera filtering conducted in conventional analyses (in our case removing two thirds of the sequences) may also remove true sequences. We found recent evidence of this when one of the most important phylogroups in the human infant gut was filtered as a chimeric sequence (1).

We believe that the risks of erroneous assignments for poorly characterized phylotypes are lower using our *de novo* semi-alignment approach. The challenge with the OTN approach, on the other hand, is that relatively long sequence reads are needed to avoid the loss of phylogenetic signals. In our case, this could have led to the relatively low bootstrap values in the OTN-based phylogenetic reconstruction. With the increased read-lengths, all next generation sequencing techniques in the range 500–1,000 nt, and the promise of long-read single molecule sequencing (5), this would probably improve the performance of the OTN approach in the future for next generation sequencing. Long-range semi alignments could potentially also be improved by utilizing conserved sequence regions to correct for long-range shifts sequence lengths due to insertions or deletions.

Compared with the previously developed multimer-based AIBIMM approach (12), phylogenetic information can be directly derived from the OTN patterns based on simple assumptions about the mutation rate and generation time. For AIBIMM or other multimer-based approaches, on the other hand, there are no underlying biological models explaining the clustering patterns; therefore, these cannot be used to directly deduce phylogenetic relations.

Different criteria for distance calculations and species clusters definition render direct comparison of the OTN and alignment-based approaches difficult. However, the fact that the OTN approach gave two peaks for pairwise differences, as compared to one peak for conventional alignment, suggests higher resolution for the OTNs, while for the traditional alignment the two peaks are merged due to noise. Even more compelling was the comparison of the phylum level taxonomic assignments in the PCA coordinate system. Here there were major differences between the OTN and conventional alignments, suggesting that conventional alignments resolve the phylum level poorly, while the OTNs enabled the capture of the majority of phylum level information.

## Figures and Tables

**Fig. 1:**
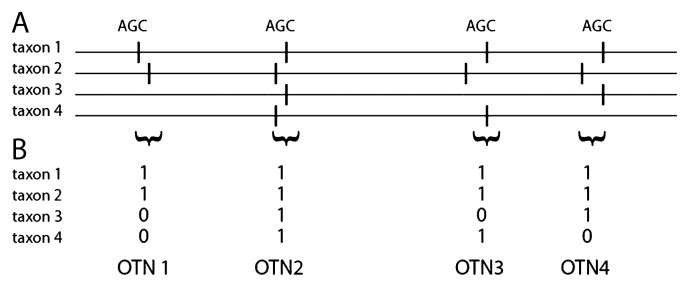
Schematic outline of the OTN identification approach. (A) Trinucleotides in the same sequence regions are considered as OTNs. (B) A table is generated for the presence/absence of the OTNs in each taxon. This table can be used for further alignment-independent analyses of the relatedness between the taxa.

**Table 1:** Alpha diversity measurements for the libraries analyzed.

Earthworm	Shannon-Wiener (H)		Simpson’s (D)	
	
	OTN^1^	OTU^2^	OTN^1^	OTU^2^
+	5.49	5.12	120.2	93.03
+	5.41	4.97	94.68	70.55
+	5.48	5.03	117.6	71.63

−	5.61	5.13	143.3	94.92
−	5.55	5.21	147.1	104.83
−	5.52	5.29	151.5	131.55

1 OTN diversity is based on complete matches.

2 OTU diversity is based on 100% identity threshold.
